# Exploring Smad5: a review to pave the way for a deeper understanding of the pathobiology of common respiratory diseases

**DOI:** 10.1186/s10020-024-00961-1

**Published:** 2024-11-22

**Authors:** Zeqiang Lin, Jiayu Zhuang, Lixia He, Siyuan Zhu, Weiguo Kong, Wenju Lu, Zili Zhang

**Affiliations:** 1 State Key Laboratory of Respiratory Disease, National Clinical Research Center for Respiratory Disease, Guangdong Key Laboratory of Vascular Disease, Guangzhou Institute of Respiratory Health, The First Affiliated Hospital of Guangzhou Medical University, Guangzhou, Guangdong China; 2 Guangzhou Medical University, Guangzhou, Guangdong China

**Keywords:** Smad5, TGF-beta superfamily, Chronic obstructive pulmonary disease, Pulmonary hypertension, Idiopathic pulmonary fibrosis

## Abstract

Smad5 (small mothers against decapentaplegic 5) protein is a receptor-regulated member of the Smad family proteins, mainly participating in the bone morphogenetic protein (BMP) signaling pathway in its phosphorylated form. This article will provide a detailed review of Smad5, focusing on its gene characteristics, protein structure, and subcellular localization properties. We will also explore the related signaling pathways and the mechanisms of Smad5 in respiratory diseases, including chronic obstructive pulmonary disease (COPD), bronchial asthma, pulmonary arterial hypertension(PAH), lung cancer, and idiopathic pulmonary fibrosis (IPF). Additionally, the review will cover aspects such as proliferation, differentiation, apoptosis, anti-fibrosis, and mitochondrial function metabolism. In addition, the review will cover aspects of proliferation, differentiation, apoptosis, anti-fibrosis and functional mitochondrial metabolism related to the above topics. Numerous studies suggest that Smad5 may play a unique and important role in the pathogenesis of respiratory system diseases. However, in previous research, Smad5 was mainly used to broadly determine the activation of the BMP signaling pathway, and its own function has not been given much attention. It is worth noting that Smad5 has distinct nuclear-cytoplasmic distribution characteristics different from Smad1 and Smad8. It can undergo significant nuclear-cytoplasmic shuttling when intracellular pH (pHi) changes, playing important roles in both the classical BMP signaling pathway and non-BMP signaling pathways. Given that Smad5 can move intracellularly in response to changes in physicochemical properties, its cellular localization may play a crucial role in the development of respiratory diseases. This article will explore the possibility that its distribution characteristics may be an important factor that is easily overlooked and not adequately considered in disease research.

## Introduction

Smad5 possesses different nucleoplasmic distribution characteristics compared to Smad1 and Smad8. It can undergo significant nucleoplasmic shuttling upon changes in intracellular pH (pHi). Additionally, it plays an important role in both canonical BMP signaling pathways and non-BMP signaling pathways. In this paper, we will provide a detailed review of the genetic features, protein structure, subcellular localization properties, and related signaling pathways of Smad5, and explore the possibility that its distributional characteristics may be an important factor that is easily overlooked and lacks consideration in disease research.

In human organisms, Smads function as signal transducers for the transforming growth factor β (TGF-β) family members (Hill 1999). TGF-β family plays a crucial role in signal transduction, regulating biological processes such as gene expression, cell proliferation, differentiation, and apoptosis (Massagué 2000). There are eight Smad family members in mammals, which are classified into three classes based on functional and structural differences (Massagué 2000). (1) The receptor-regulated Smads (R-Smads), including Smad1, Smad2, Smad3, Smad5, and Smad8/9, primarily engage in downstream signaling regulation via phosphorylation and complex formation. (2) The common mediator Smads (Co-Smads), including Smad4, neither bind to type I receptors nor are phosphorylated by them, but they form heterooligomeric complexes with activated R-Smads, which appear to be crucial for R-Smads function. (3) The inhibitory Smads (I-Smads) include Smad6 and Smad7, which antagonise the activity of the Co-Smad/R-Smad complexes. Smad2 and Smad3 are R-Smads, specifically activated by TGF-β and activin receptors. However, Smad1, Smad5, and Smad8 are R-Smads activated by BMP receptors (Massagué 2000).

Both TGFβ signaling pathway and BMP signaling pathway transduce signals through the TGF-β receptor family, which is divided into 2 subfamilies: type I receptors and type II receptors (Van Caam et al. 2017). In the canonical Smad signaling pathway, TGF-β or BMP must be activated to engage with a tetrameric receptor complex composed of receptors I and II. Type II receptors, equipped with an intracellular kinase domain, recognize TGF-β or BMP, recruit and phosphorylate the GS sequence (a Gly/Ser-rich sequence) of type I receptors. The activated type II receptors promote the formation of a heterotetramer and phosphorylate type I receptors. Subsequently, the activated type I receptors phosphorylate R-Smads, which then bind to Co-Smads (Smad4) to form a trimeric complex. This trimeric complex transloc to the nucleus to function as a transcription factor, regulating gene expression from embryonic development to adult physiological processes (Chai et al. 2015; Orlowski 2017; Van Caam et al. 2017).

## Smad5 gene characteristics and protein structure

**Fig. 1:**
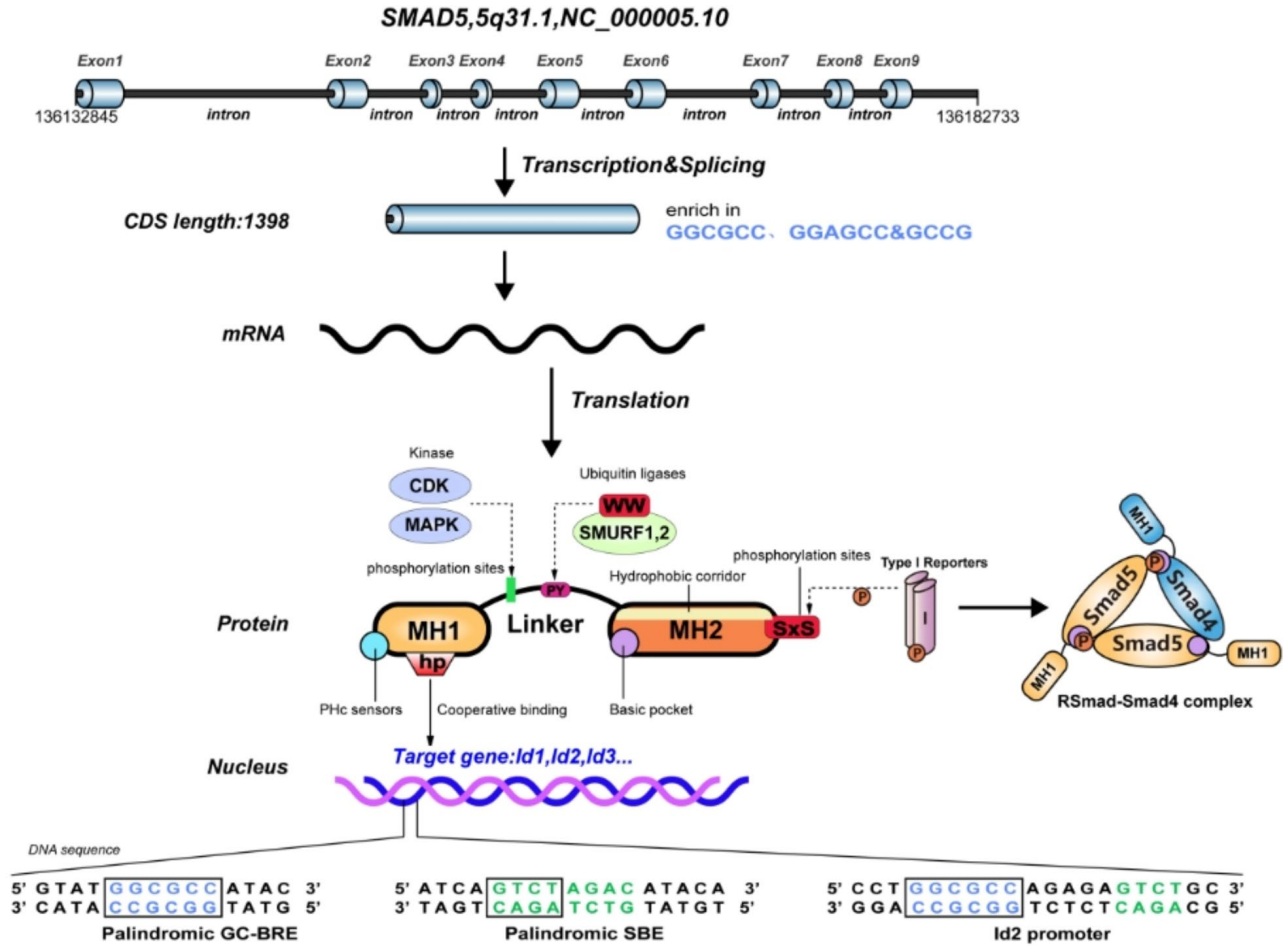
Smad5 gene structure and protein structure diagram. The Smad5 gene is located on human chromosome 5q31.1, with its binding region enriched with GGCGCC, GGAGCC, and GCCG sequences. The Smad5 protein comprises the MH1 and MH2 structural domains, as well as a Linker region. 1. MH1: Specifically binds to GC-BRE motifs and SBE motifs of DNA. The N-terminal PHc sensors are sensitive to fluctuations in intracellular pH. 2. Linker: SMURF1 and SMURF2 interact with the PY motif of phosphorylated Smad5 through the WW domains to ubiquitinate Smad5 for degradation. 3. MH2: Hydrophobic corridor mediates interactions with DNA-binding cofactors. The Ser-X-Ser motif at the C-terminal end can be phosphorylated by the type I receptors into the pS-X-pS motif. Two pS-X-pS motifs can interact with the Basic pocket, forming the RSmad-Smad4 complex

The Smad5 gene is localized to human chromosome 5q31.1 (chromosome 5, long arm, region 3, band 1, subband 1). Its genomic coordinates on the reference sequence NC_000005.10 are from base pair 136,132,845 to base pair 136,182,733. The binding region contains enriched GGCGCC, GGAGCC, and GCCG sequences, with an exon count of 9. The Smad5 protein consists of two conserved domains: the Mad homolog domain 1 (MH1) and the Mad homolog domain 2 (MH2), as well as a proline-rich variable linker region. (1) The MH1 domain is stabilized by cohesive zinc atoms, including conserved β-hairpin structure that directly binds to DNA. The Smad5 binding motif includes two types of DNA sequences: the 5′-GGC-3′ (GC-rich sequence) and 5′-GTCT-3′ (the Smad binding element, SBE), which specifically bind to the GC-BRE motif and SBE motif of DNA (Chai et al. 2015). The MH1 domain is located at the N-terminus of Smad5 and contains its unique pH sensor, which consists of a cluster of one basic (lysine) and two acidic (aspartic acid and glutamic acid) amino acids (Orlowski 2017). This sensor is sensitive to fluctuations in intracellular pH. It facilitates the translocation of Smad5 from the nucleus to the cytoplasm under alkaline conditions and promotes its interaction with hexokinase 1 (HK1) to regulate glycolysis (Fang et al. 2017). (2) The linker region, located after the MH1 domain, is a flexible fragment. It contains PPXY (PY) motifs and multiple phosphorylation sites for MAPK, CDK and other protein kinases. The PY motif is primarily recognized with the WW domain in the E3 ubiquitin ligase, such as the Smadubiquitination regulatory factor (SMURF). SMURF1 and SMURF2 interact with phosphorylated Smad5 protein through their WW domains and ubiquitinate it for degradation (Chong et al. 2010). (3) The MH2 domain is often responsible for protein–protein interactions. A continuous set of hydrophobic patches on its surface, called the Hydrophobic corridor, mediates interactions with DNA-binding cofactors. The MH2 domain is located at the C-terminus of Smad5 and contains two serine residues, called Ser-X-Ser motif, which can be phosphorylated to pS-X-pS motif by activated type I receptors (Macias et al. 2015). Two pS-X-pS motifs interact with the Basic pocket in the MH2 domain to form the RSmads-Smad4 complex **(**Fig. 1**)**.

The MH1 domain of Smad5 is enriched with sequence-binding sites for GC. The BMP-responsive Smad1 and Smad5 exhibit different DNA-binding patterns compared to TGF-β-responsive Smad3 (Morikawa et al. 2011). In addition, the MH1 domain of Smad5 displays a much stronger cooperative binding to the GC-BRE sequence than to the palindromic SBE sequence, forming a constitutive dimer on the GC-BRE sequence (Morikawa et al. 2011). The Smad5 structural domain has some differences from the other R-Smad structural domains, and these differences result in a specific biological function of Smad5 in the BMP signaling pathway.

## Cellular and tissue distribution of Smad5

In the basal state, Smad proteins can shuttle between the cytoplasm and the nucleus, thanks to their own nuclear localization signal (NLS) and nuclear export signal (NES) (Xiao et al. 2000, 2001, 2003). Nevertheless, Smad2, Smad4 and Smad8 are mainly distributed in the cytoplasm, while Smad1 and Smad3 are mainly distributed in the nucleus (Liu et al 2016). Under basal conditions, Smad2 and Smad4 are unable to accumulate within the nucleus due to their higher nuclear export efficiency relative to nuclear import (Li et al. 2018). Notably, Smad5 is unique in this respect. Unphosphorylated Smad5 is able to dynamically alter its distribution in the nucleus and cytoplasm in response to fluctuations in ambient temperature, extracellular pH (pHe), and osmotic pressure (Fang et al. 2017). In the nucleus, Smad5 mainly plays a role in BMP signaling in phosphorylated form. When Smad5 is activated and phosphorylated by the type I receptors (ALK1, 2, 3, 6) of the BMP signaling pathway, P-Smad5 binds to Smad4 and translocates to the nucleus. Once in the nucleus, P-Smad5 can interact with various transcription factors or auxiliary proteins to regulate the expression of different target genes. In the cytoplasm, unphosphorylated Smad5 plays a very important role in the regulation of cellular bioenergetic homeostasis. Temperature, cytoplasmic pH (pHc), and osmotic pressure fluctuations ultimately converge to changes in intracellular pH (pHi), and low temperatures, elevated pHc, and increased osmotic pressure alkalize the cytoplasm and cause an increase in pHi. The pHc receptors in the MH1 domain of Smad5 are very sensitive to changes in pHi, and an increase in pHi promotes the translocation of Smad5 from the nucleus to the cytoplasm (Fang et al. 2024). It has been found that Smad5 exhibits cytoplasmic accumulation when the alkaline amino acid cluster of pHc receptors in the MH1 domain is mutated to neutral amino acid, and exhibits nuclear accumulation when the acidic amino acid cluster is mutated to neutral amino acid (Fang et al. 2017). Among them, Smad5 localized in the cytoplasm significantly affects mitochondrial morphology and neural differentiation of embryonic stem cells (Fang et al. 2017).

Smad5 is primarily expressed in mesenchymal and somatic cells during embryogenesis and is found in various tissues in adults. With low tissue specificity, Smad5 is distributed across as many as 25 tissues (Fagerberg et al. 2014), including the liver, kidneys, lungs (The Tabula Sapiens Consortium* et al. 2022), and myocardium. Smad5 RNA is most highly expressed in epithelial tissue, followed by ovary, endometrium (Ulrich et al. 2022; Wang et al. 2020), and placenta. Smad5 is abundantly expressed in epithelial cells (Uhlén et al. 2015).

## Smad5-related signaling pathways

**Fig. 2:**
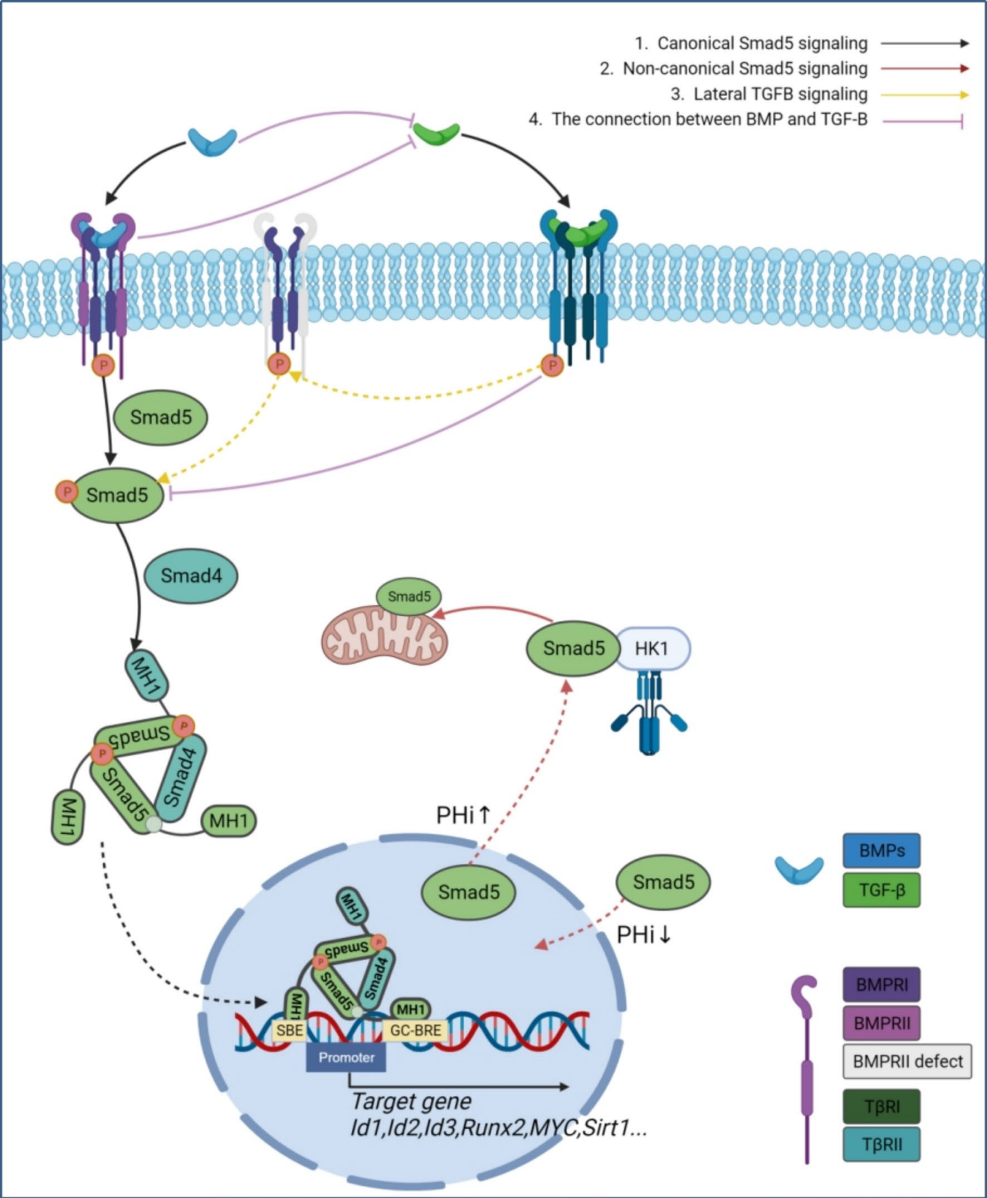
Smad5 Signal Path Diagram. 1. Canonical Smad5 signaling: BMP binds to BMPRI and BMPRII, and activated BMPRI recruits and phosphorylates Smad5. Phosphorylated Smad5s then bind to Smad4, forming a trimeric complex that moves into the nucleus. The Smad5-MH1 complex activates transcription by binding directly to GC-BRE and SBE motifs in target gene promoters. 2. Non-canonical Smad5 signaling: When intracellular pH (pHi) increases, unphosphorylated Smad5 accumulates in the cytoplasm. The alkaline pHi enhances Smad5 binding to HK1 (hexokinase 1), which boosts glycolysis. 3. Lateral TGFβ signaling: When BMPRII is defective, TGF-β binds to TβRII, leading to the phosphorylation of TβRI. This phosphorylation can then laterally induce to mediate the phosphorylation of the type I receptor in the BMP signaling pathway, regulating downstream Smad5 activation

Smad5 is functionally unique because of its protein structure. Smad5-mediated signaling pathways include the canonical BMP-Smad1/5/8 signaling pathway, non-canonical Smad5 signaling pathway, and lateral TGF-β signaling pathway. (1) In the canonical BMP-Smad1/5/8 signaling pathway, TGF-β superfamily member ligands include BMPs, AMH (anti-mullerian hormone), GDFs (growth and differentiation factors), and an activated heterotetrameric receptor complex formed by the binding of Activin A to its cognate receptor kinase. This heterotetrameric receptor complex consists of two type I receptors, including ALK1, ALK2 (ACVR2), ALK3 (BMPR IA), ALK6 (BMPR IB), and two type II receptors, including BMPR II, AMHR II, ActR IIA, and ActR IIB (Activin A Receptor Type IIA, Type IIB) (Katagiri and Watabe 2016). The activated type I receptor recruits and phosphorylates Smad1/5/8 on two Ser residues. whereas the two phosphorylated Smad1/5/8 and Smad4 bind to the Basic pocket via the pS-X-pS motif, assembling a trimeric complex and translocating into the nucleus. Smad5-MH1 in the complex activates the transcriptional regulatory machinery through direct binding to 5′-GGCGCC-3′ (GC-BRE motif) and 5′-GTCT-3′ (SBE motif) of the target genes (Id1, Id2, Id3…) promoter. (2) In the non-canonical Smad5 signaling pathway, unphosphorylated Smad5 also plays a role in cytoplasmic energy metabolism (Orlowski 2017). The pHc increases dramatically when changing physicochemical properties such as temperature, pHe and osmolality, leading to a net accumulation of unphosphorylated Smad5 in the cytoplasm. The more alkaline pHc promotes Smad5 binding to HK1, thereby increasing the rate of glycolysis (Orlowski 2017). HK1 is a rate-limiting enzyme of glycolysis and a direct binding partner of Smad5, independent of Smad1 or Smad8 (Orlowski 2017). (3) The canonical TGF-β signaling pathway mainly involves the TβRII-TβRI-Smad2/3 signaling axis. However, when BMPRII deficiency causes pulmonary arterial hypertension (PAH), TGF-β can activate lateral signaling pathways in endothelial cells (Hiepen et al. 2019). Upon binding of TGF-β to its receptor TβRII, the phosphorylated type I receptor ALK5 induces downstream Smad2/3 phosphorylation. Additionally, it mediates phosphorylation of type I receptors ALK1 and ALK2/3 in the BMP signaling pathway through lateral induction. This process subsequently regulates downstream Smad1/5/8 activation (Fig. 2).

## Smad5 and chronic obstructive pulmonary disease (COPD)

### Canonical Smad5 pathway is associated with airway remodeling and lung inflammation in COPD

Airway remodeling in chronic obstructive pulmonary disease (COPD) originates from smoking-induced changes in airway basal stem/progenitor cells (BCs). Wulin Zuo et al. found that BMP4 was highly expressed in the airway epithelium of smokers and COPD patients, mediating the transdifferentiation of normal BCs into abnormal squamous cells through activation of the BMPRIA-Smad1/5/8 signaling pathway (Zuo et al. 2019). As the primary receptor for BMP4, BMPR1A (ALK3) is specifically expressed in a subpopulation of BCs. Although the downstream Smad signaling of BMPR1A lacks tissue or cell specificity, the specific expression of BMPR1A highlights the important role of Smad in this process. Zhigang Wang et al. also found that macrophage activation had the same role in promoting airway remodeling by secreting BMP2 (Wang et al. 2021). In contrast to BMP4, BMP6 positivity is localized in airway smooth muscle cells, endothelial cells and lung macrophages, and is less expressed in airway epithelial cells. In the lung tissues of COPD patients, mRNA and protein levels of BMP6 are significantly reduced, contributing to iron deposition that induces oxidative stress, leading to tissue damage and lung inflammation (Verhamme et al. 2019). The primary reason for the two different outcomes is the varying expression and distribution of BMP ligands and their corresponding receptors, which result in BMPs often playing different roles even though they have the same signaling molecules.

### Non-canonical Smad5 pathway may be associated with enhanced glycolysis in COPD

Under aerobic conditions, the tricarboxylic acid cycle (TCA cycle) can prevent the accumulation of pyruvate. However, under hypoxic conditions, pyruvate is irreversibly converted to lactate via anaerobic glycolysis. Lactate formation limits the efficiency of energy production in the TCA cycle, which leads to an insufficient intracellular supply of adenosine triphosphate (ATP) (Maltais et al. 1996). As the severity of the disease increases according to the GOLD classification, levels of glycolytic products such as pyruvate and lactate tend to rise, while the efficiency of energy supply decreases (Xue et al. 2021). In patients with acute exacerbation of COPD (AECOPD), the levels of glycolytic metabolites are still at a higher level after the treatment, and this inefficiency of the energy supply pathway will further promote lung injury (Xue et al. 2021). Enhanced glycolysis has been found in blood samples, skeletal muscle, and mononuclear macrophages (Ryan et al. 2023) from COPD patients. Several studies in metabolomics of blood samples have revealed that aerobic and anaerobic energy metabolism balance is significantly altered in patients with COPD, correlating with glycolysis. COPD-associated skeletal muscle dysfunction is one of the factors contributing to restrictive ventilatory dysfunction, which is caused by the interaction of intrinsic factors such as oxidative stress and mitochondrial dysfunction, and extrinsic factors such as excess lactate secretion and reduced exercise tolerance (Jaitovich and Barreiro 2018). Skeletal muscle in COPD patients undergoes a shift from oxidative to glycolytic metabolism during low-level exercise or hypoxia (Maltais et al. 1996). Upregulated glycolysis in skeletal muscle of COPD patients promotes a unique post-translational glycosylation of proteins known as O-GlcNAcylation, which further shifts metabolism toward glycolysis. This causes fiber-type shifting from Type I (oxidative) muscle fibers to Type II (glycolytic) muscle fibers, leading to a reduction in protein synthesis, the loss of skeletal muscle, and even sarcopenia (Sekar and Attaway 2023).

In COPD patients with hypercapnic hypoxemia, skeletal muscle predominantly relies on anaerobic metabolism, with glycolytic pathways being preferentially activated (Fiaccadori et al. 1987). This indicates a significant disturbance in energy metabolism and acid–base balance. The function of mitochondria as the energy factories is severely compromised in this pathological context of COPD. The morphology of mitochondria in bronchial epithelial cells isolated from COPD patients is characterized by swelling, abnormal branching, and few or no cristae (Hoffmann et al. 2013). CS-exposed airway smooth muscle cells (ASMC) exhibited mitochondrial fission. There is also an increase in fission-related proteins (such as Drp1 and Fis1) and a decrease in fusion proteins (such as Mfn1/2 and OPA-1) (Aravamudan et al. 2014). Altered mitochondrial morphology weakens the structural integrity of the membrane, leading to reduced ATP production and impaired energy metabolism, forcing cells to shift from primarily oxidative phosphorylation to a more glycolysis-dependent pathway to maintain energy supply. In the pathological conditions of COPD, the mechanisms underlying the relationship among the acidic environment, mitochondrial function, and glycolysis need further investigation.

HK1 plays a key role in the conversion of oxidative phosphorylation into other metabolic pathways. And its product, glucose-6-phosphate (G6P), can be used to generate anabolic intermediates via the glycolytic pathway or the pentose phosphate pathway (PPP). The buildup of these products is detrimental to oxidative phosphorylation in normal cells. HK1 has been shown to regulate mitochondrial energy metabolism through the formation of a ring structure. HK1 rings promote the conversion of glutamine and increase the activity of TCA, thereby increasing mitochondrial energy production and decreasing pyruvate accumulation (Pilic et al. 2024). Disassembly of the HK1 ring has been associated with mitochondrial fission (Pilic et al. 2024), which is often accompanied by a metabolic shift from oxidative phosphorylation to aerobic glycolysis. Mitochondrial fission can also lead to a decrease in mitochondrial oxidative phosphorylation by causing a disruption of the mitochondrial membrane potential (Westermann 2012). HK1 is predominantly distributed in mitochondria, and the N-terminus contains the mitochondrial binding domain (MBD) of the outer mitochondrial membrane (OMM) (Pusec et al. 2019). Recent studies have shown that lack of MBD dissociates HK1 from mitochondria, leading to diminished GAPDH activity and conversion of glucose flux to PPP, which increases the release of inflammatory cytokines (De Jesus et al. 2022). In conditions associated with low-grade inflammation, such as aging and diabetes, chronic HK1 dislocation from the mitochondria may be involved in the increased inflammation (De Jesus et al. 2022; Fang et al. 2024). Selective regulation of glucose metabolism pathways by HK1 is dependent on its localization between the cytoplasm and the mitochondria and is independent of its enzymatic activity. Interestingly, Fang’s study found that Smad5 KO cells had reduced glycolytic intermediates and oxygen consumption rates (OCR). Additionally, Smad5 KO cells caused dysfunctional mitochondrial morphology and function. Immunoprecipitation identified 67 partners that bind to Smad5, and mass spectrometry confirmed these partners, which included HK1. The binding of Smad5 and HK1 increased significantly with elevated pHe. In Smad5 KO cells,the enzymatic activity of HK1 decreased and did not recover with pHe alkalinization (Fang et al. 2017). These results suggest that Smad5 maintains bioenergetic homeostasis through its interaction with HK1, and its absence negatively impacts glycolysis and mitochondrial function. Developing strategies to target Smad5 and maintain HK1 on mitochondria may help reduce inflammation and alleviate the negative sequelae of COPD and other inflammation-related diseases.

## Smad5 and pulmonary arterial hypertension

Pulmonary arterial hypertension (PAH) is a complex and progressive disease characterized by elevated pulmonary vascular resistance (PVR), mainly due to persistent vasoconstriction, homocardial vascular wall thickening and occlusive endothelial lesions. Homocardial pulmonary vessel wall thickening is mainly caused by increased proliferation and decreased apoptosis of pulmonary artery smooth muscle cells (PASMCs), along with increased proliferation and apoptosis of pulmonary artery endothelial cells (PAECs) (Sakao et al. 2009).

A member of the TGF-β superfamily, the BMPR II signaling axis is a pathway essential for pulmonary vascular homeostasis. In the 20 years since the first discovery of BMPR II deficiency associated with the development of PAH, there has been a consensus on the importance of BMPR II deficiency in the pathogenesis of PAH. BMPR II signaling pathway activity is critical in both pulmonary arterial PAECs and PASMCs, although the two vascular cell types appear to be dependent on different ligands for BMPs and different BMPR II signaling outputs. Imbalanced signaling by the TGF-β superfamily contributes extensively to dysregulated vascular cell proliferation in PAH. Hyperactive proliferative Smad2/3 signaling, reduced BMPR II and deficient antiproliferative Smad1/5/8 signaling occur simultaneously (Andre et al. 2022). BMPs ligands signal mainly through the Smad1/5/8 canonical pathway, and the BMP-BMPRII-Smad1/5/8 pathway has a protective function that is required to prevent vascular cell proliferation and consequent pathological vascular remodeling (Southgate et al. 2020). Integration of genomic data by Mendelian randomisation analysis revealed that genetic variants associated with lower expression levels of Smad5 may increase susceptibility to PAH (Rhodes et al. 2020). It has been discovered that the gene sequence of SEMA 3G contains a binding site for the Smad5 transcription factor. BMP9 can exert a protective effect against PAH by regulating SEMA3G and inhibiting the migration and network formation of pulmonary vascular endothelial cells. This process requires the involvement of ALK 1, BMPR2, and ACTR 2A. Smad 1/5 and Smad 4 knockdown attenuates this induction process (Mirza et al. 2024). However, selective loss of BMP9 has been shown to partially prevent experimental PAH. BMP9 can influence the balance of potent vasodilatory factors such as apelin and adrenomedullin (ADM), and the vasoconstrictor endothelin-1 (ET-1), through both Smad1/5/8 signaling and non-Smad pathways. This regulation leads to pulmonary vasoconstriction and pulmonary vascular remodeling (Tu et al. 2019). Inhibition of BMP9 can downregulate Smad1/5 via ALK-1 thereby downregulating ATOH8, promoting HIF-2α expression and exacerbating hypoxia in pulmonary hypertension (Morikawa et al. 2019). TGF-β and activin-like ligands primarily signal through Smad2/3. In patients with PAH, high levels of TGF-β are detected in remodeled distal small arteries and blood. TGF-β inhibits apoptosis of PASMCs through the non-canonical PI3K/AKT pathway and also promotes proliferation of PASMCs through the non-canonical PTEN-dependent pathway (Morrell et al. 2019). In vitro experiments have shown that BMPR II-deficient PAECs can acquire a Smad1/5 response to TGF-β through lateral signaling (Hiepen et al. 2019). In endothelial cells, TGF-β can additionally activate Smad1 and Smad5 (Daly et al. 2008). There may be additional levels of signaling complexity in the tissue environment (Fig. 2).

In PAH, mutations or knockdown of BMPR II in normoxia lead to significant mitochondrial dysfunction in endothelial cells, which increased regulators of mitochondrial biogenesis (p53, PGCα, TFAM), mitochondrial membrane potential, ATP production, and glycolysis, and induced mitochondrial fission and a pro-inflammatory state. This impaired the cells’ ability to efficiently generate energy and survive under stress conditions like hypoxia (Diebold et al. 2015). In parallel, transcriptomic and metabolomic analyses of human pulmonary microvascular endothelial cells with BMPR II mutations revealed extensive metabolic reprogramming. These cells showed increased aerobic glycolysis and upregulation of the PPP, along with a marked decrease in carnitine shuttle activity and fatty acid oxidation (FAO), as well as severe impairment of the TCA cycle. This TCA cycle disruption contributes further to mitochondrial dysfunction. Increased isocitrate dehydrogenase (IDH) activity was observed in both mutant cells and the serum of PAH patients (Fessel et al. 2012). These findings emphasise the role of metabolic alterations in PAH pathogenesis.

Right ventricular (RV) function is the primary determinant of survival in patients with PAH. BMPR II mutations are associated with abnormal calcium handling and altered lipid metabolism in cardiomyocytes. Additionally, enhancing recombinant BMP6 has been found to directly reduce cardiac fibrosis in the left ventricle (Boehm et al. 2021). In preclinical murine models, treatment with BMP7 stimulates the proliferation of cardiomyocytes in adulthood, and is more effective even after myocardial infarction. BMP7 stimulated proliferation through BMPRIA/ACVRI and ACVRIIA/BMPRII receptors and downstream Smad5, ERK, and AKT signaling (Bongiovanni et al. 2024). In cardiomyocytes, the deletion of Smad5 resulted in an enlargement of the left ventricular internal diameter, a reduction in fractional shortening, diminished cardiac contractility, compromised performance in treadmill tests, and a marked decrease in the fractional shortening of isolated cardiomyocytes (Umans et al. 2007). The lack of the Smad5 gene resulted in apoptosis of cardiac myocytes in vivo. The Smad5-mediated TGF-βsignals may protect cardiomyocytes from apoptosis by maintaining the integrity of the mitochondria, probably through suppression of p53 mediated pathways (Sun et al. 2005). These studies suggest that targeting Smad5 may offer a potential therapeutic approach for the combined treatment of respiratory and cardiovascular diseases.

Over the past 20 years, many approaches have been evaluated in PAH models to promote Smad1/5/8 pathway signaling in the pulmonary vasculature system to help restore PAH caused by BMPR II deficiency. These include: (1) Preventing methylation of the BMPR II promoter region: Overexpression of Switch-Independent 3a (SIN3a) inhibited the proliferation of human PASMCs and upregulated BMPR II expression. Aerosolized lung-targeted gene transfer of adeno-associated virus serotype 1 encoding human SIN3a reverses and prevents the PAH phenotype in preclinical animal models (Bisserier et al. 2021). (2) Exogenous stimulation to increased BMPR II expression: 6-mercaptopurine(6-MP) increased BMPR II expression and enhanced p-Smad1/5/8 level by activating Nur77 (nuclear receptor subfamily 4 group A member 1 (NR4A1)). In the rat model of severe PAH, 6-MP prevented and reversed abnormal vascular remodeling and RV hypertrophy. It also increased the expression of Id1 and Id3 (Kurakula et al. 2019). (3) Exogenously supplemented BMP ligands: BMP9 has been shown to reverse PAH in several models, including BMPRII^+/R899X^ mice, monocrotaline-induced PAH, and Sugen-hypoxia-induced PAH. In PAECs, BMP9 induced robust phosphorylation of Smad1/5/8 and upregulated Id1 and Id3 (Long et al. 2015). From another perspective, the choice of BMP9 may be partly due to the fact that BMP9 binds to ActRIIB with a 30-fold higher affinity than to BMPRII (Townson et al. 2012). Ex vivo stimulation of rat pulmonary arteries with BMP9 led to Smad1/5 phosphorylation exclusively in the endothelium.Not only that, BMP9 also significantly increased BMPR2 gene expression (Long et al. 2015). (4) Re-balancing TGFβ/BMP signaling: Tacrolimus (FK506) is currently investigated in ongoing Phase IIa Clinical Trials for its therapeutic effects on PAH. By releasing FKBP12 from type I receptors, FK506 activates downstream Smad1/5 signaling pathways, leading to the regulation of Id1 gene expression. Notably, in PAECs from patients with idiopathic PAH, low-dose FK506 was able to reverse dysfunctional BMPR II signaling. FK506 also improved RV function. FK506 required ALK1 as a BMPR II co-receptor to rebalance TGFβ/BMP signaling, thereby reducing hyperproliferation and decreasing collagen production in human cardiac fibroblasts (Boehm et al. 2021; Spiekerkoetter et al. 2013). Sotatercept is a fusion protein consisting of the Fc domain of human IgG linked to the extracellular domain of human ActRIIA, also known as ActRIIA-FC. Currently, it has achieved significant progress in Phase 3 clinical trials for the treatment of pulmonary arterial hypertension (Humber et al. 2021; Hoeper et al. 2023). Specifically, ActRIIA-FC functions as a ligand trap for selected members of the TGF-β superfamily, demonstrating high affinity for capturing GDF8, GDF10, Activin A, and Activin B, and effectively inhibiting the signaling of these molecules (Yung et al. 2020). The primary type II receptors for GDF8, GDF10, Activin A, and Activin B are ActRIIA and ActRIIB, which are also shared with BMPs. Meanwhile, their type I receptors, ALK4, ALK5, and ALK7, are shared with TGF-β (Massagué et al. 2023). The therapeutic strategy of Sotatercept aims to restore the molecular imbalance of p-Smad1/5/8 and p-Smad2/3 in both the TGF-β/activin and BMP pathways from the ligand perspective (Cascino et al. 2024).

## Smad5 and bronchial asthma

The airways of patients with asthma are characterized by chronic inflammation and remodeling of the bronchial walls. This complex process involves excessive epithelial proliferation, goblet cell and glandular hyperplasia, subepithelial collagen deposition, thickening of the reticular basement membrane, increased vascularity, and airway smooth muscle hyperplasia and hypertrophy, along with stromal changes (Savin et al. 2023). These structural changes in the airways are associated with major symptoms such as asthma, airway stenosis, airway irritability, and mucus hypersecretion, all of which significantly affect airway function.

In the asthma model of Macaca mulatta, exposure to house dust mite (HDM) allergens and ozone significantly downregulated p-Smad1/5/8 level in the airway epithelium. This phenomenon is observed alongside the nuclear translocation of BMPRIA and eosinophil-derived proteins (Lynn et al. 2016). In the murine model of allergic asthma, cigarette smoke increased the expression and activation of TGF-β and Smad3 through the activation of mast cells, which were further stimulated by autocrine or TGF-β released by other cells (Kim et al. 2011). Airway remodelling in patients with bronchial asthma is accompanied by subepithelial fibrosis, which disrupts the physiological function of the bronchial wall. Myofibroblasts from asthmatics produce large amounts of type III collagen, which is involved in the development of subepithelial fibrosis (Hastie et al. 2002). Human bronchial fibroblasts (HBFs) from asthmatics can undergo fibroblast-to-myofibroblast transition (FMT) induced by TGF-β1 in vitro. Impairment of the antifibrotic TGFβ/Smad1/5/8 pathway in HBF from asthma donors correlates with enhanced FMT. Activation of anti-fibrotic Smad1/5/8 signaling downregulates genes for pro-fibrotic factors such as α-SMA and fibronectin as a way to prevent FMT in HBF from asthma donors (Sarna et al. 2015; Wnuk et al. 2020). Smad5 has been relatively under-researched in the context of asthma. There is a lack of studies that sufficiently demonstrate Smad5’s antifibrotic properties, and the connection between Smad5 and pro-inflammatory factors is yet to be elucidated.

BMP7 is a cytokine that activates the TGF-β/Smad1/5/8 signaling pathway, while isoliquiritigenin (ISL) is a small-molecule activator of this pathway. BMP7 can antagonize TGF-β1-dependent fibroblast activation in mouse lung tissue, primarily affecting the synthesis of α-SMA. However, BMP7 requires supraphysiological doses to achieve therapeutic effects and faces challenges in clinical application, including potential side effects, rapid in vivo clearance, and uncertain administration routes. These have led researchers to pursue more effective small-molecule alternatives (Wnuk et al. 2020). ISL, at non-cytotoxic concentrations, can alleviate TGF-β1-induced differentiation of HBFs, reduce the proportion of myofibroblasts, and significantly decrease levels of α-SMA and fibronectin (Wnuk et al. 2020). Additionally, ISL demonstrates a desensitizing effect on HBFs in vitro models. ISL not only reduces IL-4 and IL-5 levels, which significantly improves lung function in patients with allergic asthma (Wnuk et al. 2020), but also increases activation of the Smad1/5/8-dependent pathway, which inhibits FMT. ISL has the possibility of becoming a promising candidate for antifibrotic therapy in asthma.

## Smad5 and lung cancer

In tumors, BMPs are aberrantly expressed and are involved in the development of malignant tumors and tumor-associated angiogenesis, such as lung, prostate and breast cancers (Ye and Jiang 2016). Smad5 targets the promoters of DNA-binding and differentiation-suppressor (Id1, Id2, Id3) proteins and regulates their transcription (Fig. 1). Id proteins are commonly overexpressed in embryonic and tumor cells, and are closely associated with tumor cell differentiation, proliferation and invasion. Langenfeld et al. (2013), Augeri et al. (2016), Newman et al. (2018) and Huang et al. (2022) reported that inhibition of the receptors for BMP and TGF-β downregulates Id1, Id2, and Id3, thereby inhibiting tumor cell growth and inducing apoptosis in tumor cells. Interestingly, exogenous overexpression of BMP9 significantly inhibited the migratory invasive ability of human lung squamous cell carcinoma. This suggests that the BMP-Smad signaling pathway plays a crucial and complex role in disease development.

Genetic variants in BMP genes have been found to be associated with cancer risk in lung cancer. In particular, mutations in the 3′-untranslated region of BMPs may significantly affect gene function, leading to cancer susceptibility. Polymorphisms in BMP components and regulatory genes may increase genetic susceptibility to lung cancer. 314 tagSNPs were selected from 18 genes (BMP2, 4, 6, 7, and 9, Smad 1, 4, 5, 6, 7, and 8, SMURF1 and 2, ACTR2, ALK2, ALK3, ALK6, and BMPR2) associated with the BMP pathway. Among these tagSNPs, there was a consistently significant association between Smad5 rs12719482 and the risk of lung cancer in the three population sources (P < 0.05). Smad5 rs12719482 polymorphism may be a potential etiologic factor for lung cancer in patients (Zhang et al. 2018).

There are also some experimental attempts to explore therapeutic strategies for lung cancer targeting the BMP signalling pathway. In the human lung cancer xenograft model, DMH1, a small molecule inhibitor of BMP signaling, markedly diminished lung cell proliferation, induced cell death, and cell migration and invasion in NSCLC cells by inhibiting p-Smad1/5/8 signaling and down-regulating Id1, Id2, and Id3 (Hao et al. 2014). Sul-CDA-0.05, a sulfated galactoglucan, can block angiogenesis and lung cancer cells growth in vitro and in vivo may by targeting BMPR IA and BMPR II and impeding the expression of BMP2 and VEGF. It also inhibited P-Smad1/5/8 signaling and Id1 (Huang et al. 2022). Tetramethylpyrazine (TMP) suppressed angiogenesis and tumor growth of lung cancer via blocking the BMP/Smad/Id1 signaling in A549 xenograft in nude mice (Jia et al. 2016). BMP2 promotes lung adenocarcinoma metastasis through BMP receptor 2-mediated Smad1/5 activation. The depletion of Smad1/5/8 or the inhibition of Smad1/5/8 by LDN193189 inhibitor significantly reduced cell migration (Wu et al. 2022). In lung cancer cells, combining inhibitions of BMP signaling (JL5 or JL189) with mitochondrial targeting agents (phenformin or Ym155) induced apoptosis-inducing factor caspase-independent cell death by hyperactivating AMPK (Mondal et al. 2022). The BMP receptor inhibitor DMH2 caused growth inhibition and cell death in lung cancer cells, but also led to an increase in Id1 expression. Inhibition of BMP signaling in lung cancer cells resulted in elevated levels of activated TGF-β activated kinase 1(TAK1) and TGF-β. TAK1 further activated pSmad-1/5 and increased Id1 expression in lung cancer cells. This suggests that Inhibitors targeting both BMP and TGF-β type I and II receptors may effectively suppress anti-apoptotic pathways, inducing cell death in lung cancer cells (Augeri et al. 2016). Targeting BMP signaling may offer a promising therapeutic strategy for the treatment of lung cancer.

## Smad5 and idiopathic pulmonary fibrosis

Idiopathic pulmonary fibrosis (IPF) is a chronic, aggressive, and devastating age-related disease of unknown origin. IPF is characterized by the activation of fibroblasts into myofibroblasts and excessive deposition of extracellular matrix, resulting in fibrous epithelium and honeycomb cysts, structural destruction of the lung, and loss of function. The median survival is 2–3 years, and the prognosis is less favorable than for some cancers. Pirfenidone and Nintedanib slow disease progression but do not clinically cure IPF (Chen et al. 2013; Rafii et al. 2013). Smad1/5/8 pathway activation has been shown to have potent antifibrotic effects in hepatic fibrosis, renal fibrosis, pulmonary fibrosis and scar tissue in animals and humans (Guan et al. 2022; Guo et al. 2017; Meng et al. 2016; Wang et al. 2019). In alveolar epithelial cells, IL-27 inhibited TGF-β1-induced epithelial-mesenchymal transition (EMT) by suppressing the phosphorylation of Smad1, Smad3, and Smad5, as well as the expression of TGF-βR1, and this inhibition was unaffected by the presence of TGF-β1 (Dong et al. 2016). Recent studies have found that BMP4 is significantly downregulated in lung tissues of IPF patients. In lung tissues, BMP4 activated Smad1/5/8 signaling, P-Smad1/5/8 trans-localized to the nucleus to regulate gene expression, and reduced bleomycin-induced activation of the Smad2/3 signaling pathway, thereby preventing fibroblast differentiation to myofibroblasts and extracellular matrix production (Guan et al. 2022). This study is important in guiding the pharmacologic treatment of IPF.

In recent years, various drugs have been developed for the treatment of pulmonary fibrosis, but currently, only nintedanib and pirfenidone have been approved by the FDA for the treatment of IPF. Pirfenidone, as a promising antifibrotic drug, has shown significant ability to inhibit the progression of fibrosis in various clinical and experimental studies, although concerns have been raised regarding its adverse drug reactions. Pirfenidone significantly reduced TGF-β1-augmented expression of α-SMA, fibronectin, and Gremlin1, and a reversed TGF-β1-dependent suppression of BMP4 (Jin et al. 2019). Most current studies have focused on the regulation of the TGF-β/Smad2/3 signalling pathway (Liu et al. 2024), the reduction of BMPs/BMPR II signaling plays a crucial role in the development of IPF (Ye et al. 2024), but the classical pathway mediated by Smad1/5/8 is key to the antifibrotic effects of BMP. Investigating the application of Smad1/5/8 in the treatment of IPF may provide important targets for the development of new therapies.

## Revisiting Smad5: potential non-canonical functions in respiratory diseases

In summary, Smad5 signaling pathway has an important role in the pathogenesis of respiratory diseases, and inhibition or over-activation of BMP signaling will lead to adverse effects. Phosphorylated Smad5 can participate in a variety of biological processes such as cell proliferation, differentiation, apoptosis, and anti-fibrosis (Table 1). In COPD, P-Smad5 mainly plays a role in promoting the abnormal squamous differentiation of airway basal cells. In bronchial asthma and pulmonary fibrosis, P-Smad5 mainly plays an anti-fibrotic role. In pulmonary arterial hypertension, P-Smad5 plays a role in inhibiting endothelial cell over-proliferation and apoptosis.

**Table 1:** Smad in the formation of respiratory diseases

Diseases	Signaling Molecules	Type II reporters	R-Smads	Target cells	Effect on target cells	Role in diseases	References
COPD	BMP2↑	BMPRII/ActRII/ActRIIB	Smad1/5/8↓	Bronchial epithelial cells	Suppressed β-tubulin-IV levels	Enhanced airway epithelial remodeling	Wang et al. 2021
	BMP4↑			Airway epithelial cells Airway basal stem cells	Induced squamous epithelial hyperplasia	Impaired mucus clearance Increased inflammation Enhanced airway remodeling	Zuo et al. 2019
	BMP6↓			Airway smooth muscle cells Airway endothelial cells Airway epithelial cells Lung macrophages	Reduced expression of Id1 and hepcidin Increased iron deposition	Enhanced oxidative stress	Verhamme et al. 2019
PAH	BMPs	BMPRII deficiency	Smad1/5/8↓	PAECs	Inhibited PAECs migration, tube formation, and proliferation	Exacerbated pulmonary artery hypoxic damage Increased pulmonary artery pressure and resistance	Spiekerkoetter et al. 2013, Boehm et al. 2021,Southgate et al. 2020
				PASMCs	Increased PASMCs proliferation and migration Decreased cell apoptosis	Vascular wall thickening Worsening of vascular remodeling and narrowing	
	BMP9↓	BMPRII/ActRII/ActRIIB		PAECs	Downregulation of ATOH8 thereby promoting HIF-2α expression: Increased apoptosis Endothelial dysfunction Abnormal angiogenesis Pro-inflammatory response Downregulation of SEMA 3 GB: Promoted migration and network formation of PAECs	Reduced gas exchange efficiency Exacerbated lung inflammation Exacerbated pulmonary artery hypoxic damage Increased pulmonary artery pressure and resistance	Mirza et al. 2024, Morikawa et al. 2019
	TGF-β↑	TβRII	Smad2/3↑	PASMCs	Increased PASMC proliferation and collagen synthesis	Vascular wall thickening Worsening of vascular remodeling and narrowing	Andre et al. 2022
Asthma	TGF-β↑	TβRII	Smad2/3↑	Human Bronchial Fibroblasts	FMT	Airway remodeling disrupts the physiological function of the bronchial wall	Sarna et al. 2015, Wnuk et al. 2020
					Reduced the competitive inhibition of TGF-β-induced FMT		
	BMP7↓	BMPRII/ActRII/ActRIIB	Smad1/5/8↓				
Lung cancer	BMPs↑	BMPRII/ActRII/ActRIIB	Smad5↑	Tumor cells	Id protein overexpression Affected cellular differentiation, proliferation and invasion	Promotion of malignant tumor development and tumor-associated angiogenesis Smad5 rs12719482 was associated with lung cancer risk (P < 0.05)	Langenfeld et al. 2013, Augeri et al. 2016, Newman et al. 2018, Huang et al. 2022, Zhang et al. 2018
IPF	TGF-β1↑	TβRII	Smad2/3↑	Fibroblasts Type II alveolar epithelial cells	Fibroblast senescence Mitochondrial damage Type II alveolar epithelial cell senescence	Progressive fibrotic remodeling	Guan et al. 2022
	BMP4 ↓	BMPRII/ActRII/ActRIIB	Smad1/5/8↓		Antagonized TGFβ1 signaling pathway		

Upward arrow indicates upregulation or increased expression; Downward arrow indicates downregulation or decreased expression

Over the past two decades, many research teams have invested a great deal of research in the BMP signaling pathway in the hope of addressing the clinical problems through basic research. However, no significant research applications have yet been achieved. The ligand and receptor of BMP signaling have different tissue specificities, and when the ligand is missing or the receptor is defective, it is hard for exogenous complementary drugs to effectively go all the way through the whole signaling pathway, making drug research challenging. In addition, while considering gap-filling options, attention should also be paid to intrinsic factors of Smad5 signaling molecules, such as nucleoplasmic distribution, trimeric complex formation, protein–protein binding, and protein-DNA binding affinity. Due to the complex mechanism of the TGF-β superfamily signaling pathway with more than 30 ligands, 5 type II receptors and 7 type I receptors, there is crosstalk between BMP and TGF-β signaling. Different BMP ligands bind with different affinities to the two pairs of type II and type I receptors, and the classes of downstream R-Smads activated by the receptors differ. This leading to the formation of trimeric complexes of downstream Smad4 in combination with two identical or different phosphorylated R-Smads. The MH1 structural domains in these different combinations of trimeric complexes will regulate the transcription of different target genes with different affinities.

Among these complex mechanisms, we found commonalities and properties of Smad5. In the canonical BMP signaling pathway, all BMP ligands are dependent on P-Smad1/5/8, although the receptors are different, with Smad5 being the most unique. It is important to note that since Smad5 has a high degree of homology with Smad1 and Smad8. On the one hand, the antibody detected high similarity within the phosphorylated motifs and was unable to distinguish between Smad1, Smad5, and Smad8; on the other hand, phosphorylated Smad1, Smad5, and Smad8 did not have the same effect in regulating gene transcription. As downstream of TGF-β superfamily signaling, different ligands also do not uniformly activate any of the R-Smads (Nickel and Mueller 2019).

In other tissues, we found evidence for the involvement of phosphorylated or unphosphorylated Smad5 as a unique player in disease development. In triple-negative breast cancer stem cells, tumor endothelial marker 8 (TEM8) is highly expressed, and no activation of other Smad proteins was observed besides Smad5. Rho-associated protein kinase 1 (ROCK1) directly phosphorylates Smad5, enhancing vasculogenic mimicry and stemness in tumor cells. This process promotes tumor growth and invasion. (Xu et al. 2021). In renal cell carcinoma, BMP-6 can induce IL-10-dependent M2 polarization of tumor-associated macrophages, a process that relies on Smad5 and STAT3. Under the induction of BMP-6 in vivo, both Smad5 and STAT3 are phosphorylated in macrophages. The interaction between Smad5 and STAT3 has been demonstrated through immunoprecipitation and confocal fluorescence microscopy (Lee et al. 2013). Changing the pHc of pancreatic β-cells facilitates the nuclear-cytoplasmic translocation of non-phosphorylated Smad5, which in turn affects insulin processing and secretion. Therefore, altering pHc or inhibiting the nuclear export of Smad5 may alleviate insulin deficiency, suggesting that Smad5 could serve as a potential therapeutic target for diabetes treatment (Fang et al. 2024). Although the current studies on Smad5 in respiratory diseases have only generalized to determine whether Smad1/5/8 are phosphorylated and activated, above three studies may provide insights into the role of Smad5 in exploring the mechanisms of respiratory diseases and the potential for targeted therapies. Patients with COPD develop carbon dioxide retention may cause respiratory acidosis, while patients with diabetes may experience metabolic acidosis due to the production of large amounts of ketone bodies from fat breakdown. Both conditions result in the accumulation of acidic substances, leading to acid–base imbalance. Meanwhile, Smad5 undergoes distribution changes in response to pHi fluctuations, which may mediate alterations in both BMP and non-BMP signaling pathways. Smad5 dynamically shuttles between the nucleus and cytoplasm during fluctuations in physicochemical properties. It is a unique character that should not be ignored. Besides, unphosphorylated Smad5 in the non-BMP signaling pathway can target a variety of mitochondrial proteins and regulate glycolysis (Fang et al. 2017). In patients with COPD, there is a shift in energy metabolism from oxidative phosphorylation to glycolysis or the PPP, and oxidative stress causes excessive intracellular production of acidic metabolites, which might lead to alterations in pHi and thus affect the nucleoplasmic distribution of Smad5. In patients with PAH, the cellular glycolytic pathway showed an enhanced Warburg effect and an enhanced PPP even under aerobic conditions. These clues suggest a potential association with the abnormal nucleoplasmic distribution of Smad5.

Despite growing evidence on the role of Smad5 in cellular processes, several critical knowledge gaps remain. First, the mechanisms through which non-phosphorylated Smad5 regulates key cellular functions, such as proliferation, apoptosis, or differentiation, are still inadequately understood. Furthermore, the interplay between Smad5 and other signaling pathways that govern lung epithelial cell functions, particularly in the context of pulmonary diseases, has yet to be fully explored. In addition, although Smad5 has been implicated in mitochondrial bioenergetics, its specific role in mitochondrial dynamics and metabolic regulation within diseases like COPD and PAH is not well characterized. Currently, there is no direct research investigating the relationship between Smad5 and pro-inflammatory cytokines in the context of asthma. Future research should focus on these areas to better understand comprehensive role of Smad5 in lung pathobiology and its potential as a therapeutic target.

## Data Availability

No datasets were generated or analysed during the current study.

## Abbreviations

Smad5: Small mothers against decapentaplegic 5
BMP: Bone morphogenetic protein
COPD: Chronic obstructive pulmonary disease
PAH: Pulmonary arterial hypertension
IPF: Idiopathic pulmonary fibrosis
pHi: Intracellular pH
TGF-β: Transforming growth factor β
R-Smads: Receptor-regulated Smads
Co-Smads: Common mediator Smads
I-Smads: Inhibitory Smads
GS sequence: Gly/Ser-rich sequence
Chromosome 5q31.1: Chromosome 5, long arm, region 3, band 1, subband 1
MH1: Mad homolog domain 1
MH2: Mad homolog domain 2
SBE: Smad binding element
HK1: Hexokinase 1
PY motifs: PPXY motifs
SMURF: Smadubiquitination regulatory factor
NLS: Nuclear localization signal
NES: Nuclear export signal
pHe: Extracellular pH
pHc: Cytoplasmic pH
AMH: Anti-mullerian hormone
GDFs: Growth and differentiation factors
ActR: Activin receptor
BCs: Basal stem/progenitor cells
TCA cycle: Tricarboxylic acid cycle
ATP: Adenosine triphosphate
AECOPD: Acute exacerbation of COPD
ASMC: Airway smooth muscle cell
G6P: Glucose-6-phosphate
PPP: Pentose phosphate pathway
MBD: Mitochondrial binding domain
OMM: Outer mitochondrial membrane
OCR: Oxygen consumption rate
PVR: Pulmonary vascular resistance
PASMCs: Pulmonary artery smooth muscle cells
PAECs: Pulmonary artery endothelial cells
ADM: Adrenomedullin
ET-1: Endothelin-1
FAO: Fatty acid oxidation
IDH: Isocitrate dehydrogenase
RV: Right ventricular
SIN3a: Switch-Independent 3a
6-MP: 6-Mercaptopurine
NR4A1: Nuclear receptor subfamily 4 group A member 1
FK506: Tacrolimus
HDM: House dust mite
HBFs: Human bronchial fibroblasts
FMT: Fibroblasts-to-myofibroblasts transition
ISL: Isoliquiritigenin
TMP: Tetramethylpyrazine
TAK1: TGF-β activated kinase 1
EMT: Epithelial-mesenchymal transition
TEM8: Tumor endothelial marker 8
ROCK1: Rho-associated protein kinase 1
